# Women’s Satisfaction With Telehealth Services During The COVID-19 Pandemic: Cross-sectional Survey Study

**DOI:** 10.2196/41356

**Published:** 2022-10-14

**Authors:** Diletta F Mittone, Caitlin P Bailey, Ebony L Eddy, Melissa A Napolitano, Amita Vyas

**Affiliations:** 1 Department of Prevention and Community Health Milken Institute School of Public Health The George Washington University Washington, DC, DC United States

**Keywords:** telehealth, COVID-19, maternal-child health, Perinatal, pediatrics, telemedicine, pregnancy, women's health, patient outcome

## Abstract

**Background:**

Since March 2020, the need to reduce patients’ exposure to COVID-19 has resulted in a large-scale pivot to telehealth service delivery. Although studies report that pregnant women have been generally satisfied with their prenatal telehealth experiences during the pandemic, less is known about telehealth satisfaction among postpartum women.

**Objective:**

This study examined telehealth satisfaction among both pregnant and recently pregnant women during the COVID-19 pandemic, to determine whether demographic factors (ie, race, age, marital status, education level, household income, and employment status) are associated with telehealth satisfaction in this population.

**Methods:**

A web-based cross-sectional survey designed to capture data on health-related behaviors and health care experiences of pregnant and recently pregnant women in the United States was disseminated in Spring 2022. Eligible participants were at least 18 years old, identified as a woman, and were currently pregnant or had been pregnant in the last 3 years.

**Results:**

In the final analytic sample of N=403, the mean telehealth satisfaction score was 3.97 (SD 0.66; score range 1-5). In adjusted linear regression models, being aged 35-44 years (vs 18-24 years), having an annual income of ≥ US $100,000 (vs < US $50,000), and being recently (vs currently) pregnant were associated with greater telehealth satisfaction (*P*≤.049).

**Conclusions:**

Although perinatal women are generally satisfied with telehealth, disparities exist. Specifically, being aged 18-24 years, having an annual income of < US $50,000, and being currently pregnant were associated with lower telehealth satisfaction. It is critical that public health policies or programs consider these factors, especially if the expanded use of telehealth is to persist beyond the pandemic.

## Introduction

The COVID-19 pandemic has profoundly impacted health care service delivery in the United States. Since March 2020, the need to reduce COVID-19 exposure for health care professionals and patients, preserve supplies of personal protective equipment, and reduce burden on health care facilities has resulted in a large-scale pivot to telehealth service delivery [1]. Telehealth is defined by the US Department of Health and Human Services as the delivery of health care services without an in-person office visit and primarily through internet access on a computer, tablet, or smartphone [2]. Before the pandemic, telehealth comprised less than 1% of outpatient visits, but this number rose to 13% in the early months of the pandemic [3]. In fact, telehealth visits increased by 154% in the last week of March 2020, when the pandemic was intensifying in the United States, compared to the same period in 2019 [4]. This surge in the use of telehealth services continued throughout 2020 with the number of telehealth visits made by Medicare patients increasing 63-fold from 840,000 in 2019 to nearly 52.7 million in 2020 [5]. Although the use of telehealth has receded since its peak in 2020, it remains high comprising 8% of outpatient visits in 2021 [3].

Given the rise in telehealth and the sustained use of these services throughout the pandemic, it is important to examine the experiences of patients using these services in order to understand challenges and address gaps with this health care delivery model. It is especially important to understand the unique experiences of women seeking prenatal and postnatal care through telehealth services because these types of maternal health services are critical to ensuring the health and safety of women and their children. Broadly, patients have reported positive experiences and high satisfaction with telehealth services during the pandemic [6,7]. However, the telehealth experiences among prenatal and postpartum women during the pandemic are not well understood, and women who have been pregnant or recently pregnant during the pandemic and used telehealth services for their routine care may have unique challenges and experiences. Although studies have found that pregnant women are generally satisfied with their prenatal telehealth experiences during the pandemic, less is known about the telehealth satisfaction of postpartum women [8-10]. Given the critical role of prenatal and postpartum health care in preventing adverse pregnancy outcomes, more research is needed to fully understand the factors (eg, age, income, and race) that impact the telehealth experiences of pregnant and recently pregnant women during the pandemic.

Prior work has identified several sociodemographic factors associated with telehealth satisfaction. Recent studies on the relationship between age and telehealth satisfaction during the pandemic have found that older people are generally less satisfied with their telehealth experience [11]. Some reasons for this may include greater challenges with technology and more concerns about privacy among older people. A study of patient satisfaction with telehealth in a rural community during the pandemic found that adults 35 years and older were less satisfied with telehealth compared to younger adults between the ages of 18 and 34 years [12]. Similarly, a study of patients in an urban community found that younger adults had more positive experiences with their telehealth visits compared to older adults [13]. Despite the consistent theme that younger patients report greater telehealth satisfaction, further research is needed to understand whether this relationship is true among women of childbearing age. In focusing on the telehealth satisfaction of pregnant and recently pregnant women, it is possible a different trend may emerge between age and telehealth satisfaction.

Income and education may also be factors related to patient telehealth satisfaction, though less is known about these relationships. A nationally representative survey of US households during the pandemic found that although telehealth use was lowest among households earning less than US $100,000 annually, telehealth satisfaction did not significantly differ by income [7]. Similarly, another study of telehealth patients did not find any significant differences in patient satisfaction by income [11]. Interestingly, a study of low-income pregnant women found that women with a yearly household income less than US $25,000 were significantly more likely to prefer a telehealth visit over an in-person visit compared to those earning more than US $25,000 annually [8]. In addition to income, more research is also needed to understand the role of education in telehealth experiences. One study found no significant differences in telehealth satisfaction by educational attainment [13]. However, another study found that patients with advanced degrees reported significantly fewer technology difficulties during a telehealth visit compared to those who reported lower educational levels [14]. Given the varied findings on how income and education are associated with telehealth satisfaction, more research is needed to elucidate these relationships, especially among pregnant and recently pregnant women of varying socioeconomic status.

Racial minority groups were disproportionately affected during the pandemic and experienced higher rates of COVID-19–related hospitalization and death as well as higher rates of job loss [15]. Given these racial disparities, it is especially important to understand the telehealth experiences of people of color. Although some research has shown greater telehealth satisfaction among non-White patients [11], other studies have found that non-White patients report poorer telehealth satisfaction [16]. Furthermore, other studies have not found any statistically significant differences in telehealth preference by race [7,13]. The lack of consistent findings on the impact of race on patient telehealth satisfaction paired together with the racial health disparities that have long existed in the United States make this a crucial gap to address if telehealth is to become an equitable health care delivery model during the pandemic and beyond.

Since the onset of the COVID-19 pandemic in mid-March 2020, the use of telehealth services has surged to unprecedented levels. Given this surge coupled with the critical role that maternal health services play in preventing adverse pregnancy outcomes for women and their newborns, it is necessary to understand the experiences of women using telehealth during the perinatal period and the different factors that may affect their satisfaction with telehealth. Specifically, it is important to examine the role of age, income, education, and race on women’s telehealth experiences, since research in this area is limited and findings are often inconsistent. By understanding the different factors that are at play when women use telehealth services, we may be able to identify and address disparities associated with this health care delivery model. For this reason, this study seeks to examine telehealth satisfaction among pregnant and recently pregnant women during the COVID-19 pandemic and to determine whether demographic factors (eg, race, age, and income) are predictive of telehealth satisfaction in this population.

## Methods

### Study Design and Sample

A web-based cross-sectional survey, built using Qualtrics software, was conducted from March 22nd to May 19th of 2022. The survey took approximately 10-15 minutes to complete, and all responses were anonymous. Eligibility criteria included adults ages 18 years and older who identify as a woman and are currently pregnant or have been pregnant in the last 3 years. Survey participants were recruited via Washington, DC prenatal clinic email listserves, social media (ie, Facebook, Instagram, and GroupMe) and Centiment, a web-based survey research company that recruits and pays individuals who meet researcher specified demographic criteria [17]. Individuals recruited by Centiment were living in DC, Maryland, or Virginia at the time of survey participation. Study procedures were approved by the first author’s institutional review board. Of the 759 current or previously pregnant participants who completed the survey, 435 (57%) reported using telehealth services since the start of the COVID-19 pandemic. Of those, 17 were missing responses to the telehealth satisfaction scale, and 15 were missing responses to demographic questions. Therefore, the final analytic sample size was N=403.

### Measures

#### Telehealth Satisfaction

Telehealth satisfaction was measured using an 11-item scale. Participants were first asked the following question: “Since the start of the COVID-19 pandemic, have you had a health care appointment using telehealth (provision of health care remotely)?” Those who answered yes were asked to respond to the following prompt: “Please rate your level of agreement with the following statements about your most recent telehealth experience.” Statements included the following: “The technology did not work for me,” “The technology was helpful in connecting me with my provider,” “I felt comfortable using telehealth,” “My doctor was attentive to me during the appointment,” “I felt my doctor was able to address my needs without a physical examination,” “I would like to continue using telehealth,” “I was satisfied with the care I received using telehealth,” “Telehealth was convenient for me,” “Telehealth made it easier for me to receive care,” “Telehealth made filling prescriptions easier,” and “I was able to receive care quickly using telehealth.” Participants responded on a 5-point Likert scale from strongly disagree (1) to strongly agree (5). Item responses were reverse coded as needed, added together, and divided by 11 to produce a total score with the possible range of 1-5.

#### Demographic Variables

Survey respondents were asked to provide demographic information including age, race, marital status, education level, annual household income, employment status, and pregnancy status (current vs recent).

### Analysis

Descriptive statistics were generated for all demographic variables of interest. Demographic characteristics were compared by race (ie, Black or African American, White, and other races) using chi-square tests. Telehealth satisfaction was also compared across demographic characteristics using ANOVA tests. Unadjusted simple regression models predicting telehealth satisfaction score from each demographic variable were tested. Finally adjusted regression models predicting telehealth satisfaction score from all demographic characteristics entered simultaneously into the model were tested. Statistical analyses were conducted using RStudio (version 1.3.1056) [18]. A significance level of <.05 was determined a priori.

### Ethics Approval

This study was approved by the George Washington University Institutional Review Board (NCR213844).

## Results

The analytical sample (N=403) was majority 25-34 years old, married or in a domestic partnership, employed full-time, and recently (as opposed to currently) pregnant. The mean telehealth satisfaction score was 3.97 (SD 0.66; score range 1-5). Table 1 presents the full demographic information for the analytic sample. Significant differences in demographic characteristics were identified by race (Black or African American, White, and other races), age category, marital status, education level, annual household income, and employment status, with Black or African American mothers tending to be younger, more often single, less educated, having lower annual household incomes, and more often unemployed (Table 1).

Significant differences in telehealth satisfaction score were identified by age category, marital status, education level, annual household income, employment status, and pregnancy status. Specifically, telehealth satisfaction score (mean 4.25, SD 0.54) was highest in participants who were aged 35-44 years, married or in a domestic partnership (mean 4.00, SD 0.62), had a master’s degree (mean 4.17, SD 0.56), had a household income of ≥ US $100,000 (mean 4.16, SD 0.50), were employed full-time (mean 4.04, SD 0.63), and were recently pregnant (mean 4.14, SD 0.63; Table 2).

In unadjusted linear regression models, age, marital status, education level, household income, employment status, and pregnancy status were associated with telehealth satisfaction. Specifically, being of older age (vs 18-24 years of age), being married or in a domestic partnership (vs single), having a bachelor’s or master’s degree (vs high school education or less), having an annual income of ≥ US $100,000 (vs < US $50,000), being employed full-time (vs part-time employment or unemployed), and being recently (vs currently) pregnant were associated with greater telehealth satisfaction (*P*≤.049; Table 3).

In adjusted linear regression models, being aged 35-44 years (vs 18-24 years), having an annual income of ≥ US $100,000 (vs < US $50,000), and being recently (vs currently) pregnant were associated with greater telehealth satisfaction (*P*≤.049; Table 3).

**Table 1:** Descriptive characteristics of the analytic sample. Statistical comparisons are conducted using chi-square tests; italicized *P* values are significant.

Characteristics		Full sample (N=403), n (%)	Black or African American (N=76), n (%)	White (N=194), n (%)	Other races (N=133), n (%)	*P* value	
**Age (years)**							*.001*
	18-24	57 (14.14)	22 (28.95)	19 (9.79)	16 (12.03)		
	25-34	261 (64.76)	44 (57.89)	130 (67.01)	87 (65.41)		
	35-44	85 (21.09)	10 (13.16)	45 (23.20)	30 (22.56)		
**Marital status**							*<.001*
	Married or domestic partnership	366 (90.82)	56 (73.68)	183 (94.33)	127 (95.49)		
	Single, divorced, or widowed	37 (9.18)	20 (26.32)	11 (5.67)	6 (4.51)		
**Highest education**							*<.001*
	High school or less	85 (21.09)	28 (36.84)	27 (13.92)	30 (22.56)		
	Associate degree or trade school	51 (12.66)	12 (15.79)	25 (12.89)	14 (10.53)		
	Bachelor’s degree	126 (31.27)	15 (19.74)	75 (38.66)	36 (27.07)		
	Master’s degree	99 (24.57)	12 (15.79)	54 (27.84)	33 (24.81)		
	Professional degree or PhD	42 (10.42)	9 (11.84)	13 (6.70)	20 (15.04)		
**Annual household income (US $)**							*<.001*
	<50,000	88 (21.84)	27 (35.53)	28 (14.43)	33 (24.81)		
	50,000-99,999	116 (28.78)	26 (34.21)	60 (30.93)	30 (22.56)		
	≥100,000	199 (49.38)	23 (30.26)	106 (54.64)	70 (52.63)		
**Employment**							*.03*
	Full-time	250 (62.03)	43 (56.58)	132 (68.04)	75 (56.39)		
	Part-time	63 (15.63)	10 (13.16)	32 (16.49)	21 (15.79)		
	Unemployed	90 (22.33)	23 (30.26)	30 (15.46)	37 (27.82)		
**Pregnancy status**							.09
	Current	179 (44.42)	33 (43.42)	77 (39.69)	69 (51.88)		
	Recent	224 (55.58)	43 (56.58)	117 (60.31)	64 (48.12)		

**Table 2:** Differences in telehealth satisfaction by demographic variables. Telehealth satisfaction possible range is 1-5; statistical comparisons were conducted using ANOVA; italicized *P* values are significant.

Characteristics			Telehealth satisfaction, mean (SD)		*P* value
**Race**					.19
	White	4.03 (0.59)			
	Black or African American	3.89 (0.76)			
	Other	3.92 (0.68)			
**Age (years)**					*<.001*
	18-24	3.66 (0.73)			
	25-34	3.94 (0.64)			
	35-44	4.25 (0.54)			
**Marital status**					*.005*
	Married or domestic partnership	4.00 (0.62)			
	Single, divorced, or widowed	3.68 (0.93)			
**Highest education**					*.001*
	High school or less	3.79 (0.76)			
	Associate degree or trade school	3.81 (0.79)			
	Bachelor’s degree	3.99 (0.55)			
	Master’s degree	4.17 (0.56)			
	Professional degree or PhD	3.95 (0.66)			
**Annual household income (US $)**					*<.001*
	<50,000	3.70 (0.74)			
	50,000-99,999	3.83 (0.72)			
	≥100,000	4.16 (0.50)			
**Employment**					*.010*
	Full-time	4.04 (0.63)			
	Part-time	3.79 (0.62)			
	Unemployed	3.88 (0.72)			
**Pregnancy status**					*<.001*
	Current	3.74 (0.62)			
	Recent	4.14 (0.63)			

**Table 3:** Linear regression predicting telehealth satisfaction from demographic variables. Italicized *P* values are significant.

Characteristics		Model 1^a^		Model 2^b^		
		*β* (95% CI)	*P* value	*β* (95% CI)	*P* value	
**Race**						
	White	—^c^	—	—	—	
	Black or African American	–.13 (–0.31 to 0.04)	.13	.03 (–0.14 to 0.20)	.71	
	Other	–.11 (–0.25 to 0.04)	.14	–.05 (–0.19 to 0.09)	.49	
**Age**						
	18-24	—	—	—	—	
	25-34	.28 (0.10 to 0.46)	*.003*	.08 (–0.10 to 0.27)	.38	
	35-44	.59 (0.38 to 0.80)	*<.001*	.23 (0.001 to 0.47)	*.049*	
**Marital status**						
	Married or in domestic partnership	—	—	—	—	
	Single, divorced, or widowed	–.32 (–0.54 to 0.10)	*.005*	–.21 (–0.44 to 0.02)	.08	
**Highest education**						
	High school or less	—	—	—	—	
	Associate degree or trade school	.02 (–0.21 to 0.24)	.88	–.07 (–0.28 to 0.14)	.52	
	Bachelor’s degree	.21 (0.03 to 0.38)	*.024*	–.04 (–0.24 to 0.15)	.66	
	Master’s degree	.38 (0.20 to 0.57)	*<.001*	–.03 (–0.25 to 0.19)	.82	
	Professional degree or PhD	.17 (–0.07 to 0.41)	.17	–.19 (–0.45 to 0.07)	.15	
**Annual household income ($ US)**						
	<50,000	—	—	—	—	
	50,000-99,999	.14 (–0.04 to 0.31)	.13	.06 (–0.13 to 0.24)	.55	
	≥100,000	.47 (0.31 to 0.63)	*<.001*	.33 (0.13 to 0.54)	*.001*	
**Employment**						
	Full-time	—	—	—	—	
	Part-time	–.25 (–0.43 to –0.07)	*.007*	–.10 (–0.28 to 0.08)	.28	
	Unemployed	–.16 (–0.31 to –0.0003)	*.049*	–.02 (–0.18 to 0.13)	.76	
**Pregnancy status**						
	Current	—	—	—	—	
	Recent	.40 (0.28 to 0.52)	*<.001*	.31 (0.19 to 0.44)	*<.001*	

^a^Unadjusted; each predictor was entered into a separate model.

^b^All covariates were entered simultaneously.

^c^Not applicable.

## Discussion

### Principal Findings

In this study, we examined how women’s satisfaction with telehealth services during the COVID-19 pandemic differed by demographic factors. Results revealed that several demographic factors were associated with greater telehealth satisfaction, including older age, being married or in a domestic partnership, higher income, having attained a bachelor’s or master’s degree, and full-time employment. However, telehealth satisfaction did not significantly differ by race, even after adjusting for other demographic factors.

Pregnant and recently pregnant women reported generally positive experiences using telehealth during the pandemic. These findings are consistent with previous literature reporting on patients’ positive experiences and high satisfaction with telehealth services during the pandemic [6,7]. Although not surprising, this finding is particularly important because it suggests that pregnant and recently pregnant women are satisfied with using telehealth services, despite the type of care that may be unique to this group, such as prenatal and postpartum care. Given that pregnant and recently pregnant women are satisfied using telehealth, it is important for health care policies and programs to consider sustaining the use of maternal telehealth services beyond the pandemic. For example, providers may consider replacing routine in-person prenatal visits with prenatal telehealth visits or using telehealth to provide postpartum lactation support and screening for postpartum depression.

Results revealed a significant association between age and telehealth satisfaction, such that older age was associated with greater telehealth satisfaction. This finding is surprising because it is contrary to much of the literature that has consistently found younger age to be associated with more positive telehealth experiences [11-13]. This may be because our sample only included women of childbearing age, which limited the range of ages represented in this study. Furthermore, it is possible that other factors affected the relationship between age and telehealth satisfaction in our sample. For example, older women may have been more likely to have older children that could have assisted them with accessing and using the telehealth platform. Alternatively, younger women may be more inexperienced with the experience of pregnancy, compared to older women, and may need more in-person support during their care [19]. Future research should investigate the relationship between age and telehealth satisfaction among women of childbearing age to identify factors that may be contributing to this unexpected relationship.

Recent studies exploring the impact of income and education on telehealth experiences have produced varied and inconsistent findings [7,8,13,14]. This study found that higher income and higher educational attainment (up to a master’s degree) were associated with greater telehealth satisfaction. These data underscore the importance of providing women from underserved communities with additional support when using telehealth services. Ensuring equitable access to telehealth services must be a priority; and public health policies or programs should implement strategies to mitigate the challenges women experience using telehealth services, particularly during the perinatal period. For example, replacing written instructions with graphics or visuals would allow women with lower literacy levels to navigate telehealth platforms more easily. Policy efforts to ensure equitable access to telehealth are crucial to eliminate disparities in patient satisfaction, especially if the current increased use of telehealth sustains beyond the pandemic.

This study did not find any significant differences in telehealth satisfaction by race. This is surprising given the health and health care access disparities that have long persisted in the United States for people of color [20]. This finding suggests that racial minority groups, such as Black women, are just as satisfied with the services they have received through telehealth as White women. However, it is also important to consider women’s access to telehealth services and potential barriers. Perhaps, women of color are satisfied with telehealth when they have access to it, but securing that access may be a greater challenge. For example, a recent survey of pregnant women living in rural areas found that although women reported a positive experience with prenatal telehealth visits overall, common barriers included poor internet and phone connectivity, childcare responsibilities, and lack of equipment [21], factors that may disproportionately impact minority groups. Although it is important to understand women’s experiences using telehealth, ensuring equitable access to these services is paramount and public health programs or policies are needed to reduce barriers to telehealth access. Given the increases we have seen in maternal morbidity or mortality in the United States [20], access to telehealth for pregnant or postpartum women could expand care and reduce maternal health disparities.

### Limitations

Although the findings from this study are important and contribute to the recent literature on patient telehealth experiences during the COVID-19 pandemic, there are certain limitations that should be noted. First, this sample of pregnant and recently pregnant women was highly educated and affluent. Nearly 70% of the sample held at least a bachelor’s degree, and almost 50% had an annual household income of at least US $100,000. It is important to note that despite the high educational attainment and income level of our sample, disparities related to income and education still emerged. This underscores the importance of ensuring equitable access to telehealth services, especially in underserved or marginalized communities. A second limitation of this study is that women were asked to report on their most recent telehealth experience and not specifically on their experience receiving prenatal or postpartum care through telehealth. For this reason, we cannot assume these findings are reflective of women’s experiences using telehealth for prenatal or postpartum care, specifically, and future research should explore this. Third, another limitation is the potential for recall bias in the sample, especially among women who were not currently pregnant and were asked to recall past telehealth experiences. Finally, this study was cross-sectional, and findings cannot be used to inform causal mechanisms. Future work is needed to identify women’s perceptions of telehealth care longitudinally. For example, studies might follow women over the course of their pregnancy to understand how their perceptions change over time.

### Conclusions

Given the increase in telehealth services since the start of the COVID-19 pandemic in March 2020, it is important to understand the experiences of women accessing these services and the different factors that impact their satisfaction with these services. Although women are generally satisfied with telehealth, there are also important disparities that exist and it is critical that public health policies or programs consider these factors, especially if the expanded use of telehealth is to persist beyond the pandemic. Ensuring equitable access to telehealth and providing tailored support to women is key to eliminating these disparities. Although patients report high satisfaction with telehealth, future studies should investigate the barriers and challenges related to telehealth access among underserved populations. Future studies should also investigate clinical outcomes related to prenatal and postpartum telehealth services, especially if telehealth is a convenient and well-regarded model for delivering these types of maternal health care services.
